# Conventional MRI‐Based Semantic Features for Differentiation of Pediatric Medulloblastoma and Ependymoma in the Fourth Ventricle: Insights From a Multi‐Center Retrospective Study

**DOI:** 10.1002/cns.70999

**Published:** 2026-06-24

**Authors:** Yu Han, Yu‐yao Wang, Yue‐wen Hao, Yi‐bin Xi, Yong‐hong Qi, Xiao‐li Du, Wen‐jun Cui, Si‐jie Xiu, Xue‐ying Zhou, Wen Wang, Wei‐chen Li, Jin Zhang

**Affiliations:** ^1^ Department of Radiology & Functional and Molecular Imaging Key Lab of Shaanxi Province, Tangdu Hospital Fourth Military Medical University Xi'an Shaanxi China; ^2^ Department of Radiology Xi'an Children's Hospital Xi'an Shaanxi China; ^3^ Department of Radiology Xi'an People's Hospital (Xi'an Fourth Hospital) Xi'an Shaanxi China; ^4^ Department of Radiology Qinghai Provincial People's Hospital Xining Qinghai China; ^5^ Department of Radiology Chengdu First People's Hospital Chengdu Sichuan China; ^6^ Department of Magnetic Resonance Lanzhou University Second Hospital Lanzhou Gansu China

**Keywords:** ependymoma, magnetic resonance imaging, medulloblastoma, semantic feature

## Abstract

**Objective:**

Differentiating pediatric medulloblastoma (MB) from ependymoma (EA) in the fourth ventricle remains challenging due to overlapping clinical and imaging features. This study aimed to identify distinctive semantic features and develop a feature‐based model for differentiating MB from EA using conventional MRI.

**Methods:**

This multi‐center retrospective MRI study enrolled 295 pediatric patients, including 184 MB and 111 EA cases, allocated to the training, internal validation set, and external testing set. Subsequently, 13 semantic features were extracted. After feature selection, quantitative parametric models and mixed parametric models were constructed and evaluated. Finally, three junior and three senior radiologists completed independent and model‐assisted assessments.

**Results:**

MB showed significantly greater left–right/upper‐lower (0.98 vs. 0.76) and anterior–posterior/upper‐lower (0.88 vs. 0.66) diameter ratios compared to EA (both *p* < 0.001). The pathognomonic “sea anemone sign” (100% specific for MB) occurred in 38.60% of MB cases. The support vector machine (SVM) model achieved optimal performance, with areas under the receiver operating characteristic curves (AUCs) of 0.946/0.921/0.915 and accuracies of 0.906/0.907/0.832 across training/internal validation/external testing sets. With SVM model assistance, diagnostic performance improved for both senior and junior radiologists across all datasets. In the external testing set, the AUCs increased to 0.849–0.893 for junior radiologists and 0.893–0.913 for senior radiologists, with the accuracies of 0.832–0.858 and 0.850–0.867, respectively.

**Conclusion:**

The sea anemone sign is highly specific for MB. The SVM model using conventional MRI semantic features achieved robust discrimination between MB and EA, significantly augmenting the diagnostic accuracy of radiologist assessment.

## Graphical abstract

The sea anemone sign is 100% specific for MB. The SVM model using conventional MRI semantic features achieved robust discrimination between MB and EA, significantly augmenting the diagnostic accuracy of radiologist assessment.

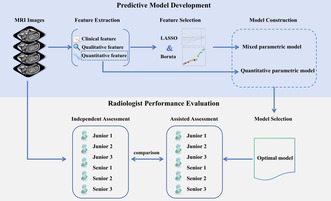

## 1 Introduction

Medulloblastoma (MB) and ependymoma (EA) are two common pediatric fourth ventricular tumors, accounting for approximately 30%–40% and 10%–15% of posterior fossa tumors in children, respectively [1, 2, 3]. Medulloblastoma, originating from the posterior medullary velum, demonstrates aggressive biological behavior with high recurrence rates, necessitating postoperative radiotherapy and chemotherapy [4, 5, 6]. In contrast, EA arises from ventricular ependymal cells, typically managed with surgery and radiotherapy, while adjuvant chemotherapy is administered when necessary, exhibiting comparatively favorable prognoses [4, 7, 8]. Therefore, precise preoperative discrimination between MB and EA holds critical clinical implications for therapeutic decision‐making and prognostic assessment.

Magnetic resonance imaging (MRI), as a non‐invasive imaging technique, plays an important role in the clinical practice of brain tumors. Advanced MRI sequences, including diffusion‐weighted imaging, magnetic resonance spectroscopy, and perfusion‐weighted imaging, have shown potential in differentiating MB from EA [9, 10, 11]. Nevertheless, the adoption of these techniques in clinical settings is hindered by additional costs, prolonged scanning time, and inconsistent diagnostic thresholds across institutions. Recent studies employing radiomics approaches have achieved promising performance in distinguishing MB from EA [10, 11, 12, 13]. However, their clinical translation faces two critical limitations: insufficient interpretability and absence of standardized implementation protocols [14, 15].

In contrast, conventional MRI‐based semantic features remain clinically prevalent due to their simplicity and strong interpretability. These features reflect macroscopic manifestations of histological differences between MB and EA, particularly in cellular density and vascular distribution patterns [4, 16, 17]. However, existing studies using basic semantic features (tumor size, signal intensity, enhancement heterogeneity) have demonstrated suboptimal diagnostic performance [18, 19, 20], which may be attributed to insufficient sample sizes and lack of pathognomonic imaging biomarkers. Meanwhile, age is critical in differentiating MB from EA, with 90% of MB cases occurring in children < 14 years of age [21]. The inclusion of the 14‐ to 18‐year‐old population in previous studies introduces a potential selection bias. Therefore, clinical practice should concentrate more on differentiating MB from EA in patients under 14 years old.

In this study, we aim to assess the semantic features of MB and EA in the fourth ventricle of children under 14 years using conventional MRI, and to develop a model for distinguishing MB from EA.

## 2 Materials and Methods

### 2.1 Patients

This study was approved by the institutional review board of Tangdu Hospital (local approval number: K‐HG‐202507‐05). Due to the retrospective nature of this study, the requirement for informed consent was waived.

**FIGURE 1:**
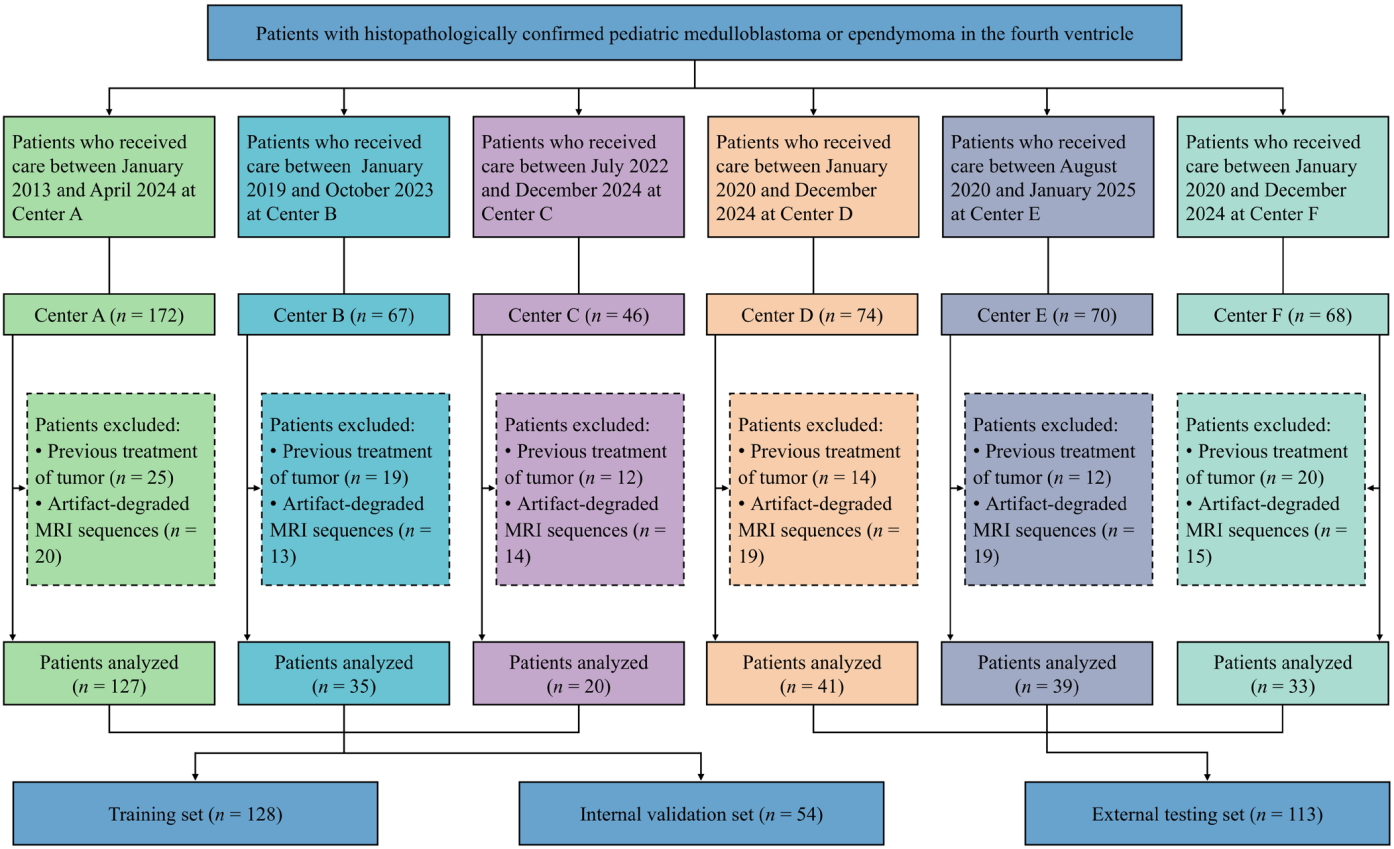
Patient selection flowchart.

Potentially eligible pediatric patients with pathologically confirmed pediatric MB and EA in the fourth ventricle from January 2013 to January 2025 at six centers were enrolled according to the following inclusion and exclusion criteria. The inclusion criteria were: (i) age ≤ 14 years; (ii) availability of preoperative MRI scans, including T1‐weighted imaging (T1WI), T2‐weighted imaging (T2WI), fluid‐attenuated inversion recovery (FLAIR), and contrast‐enhanced T1WI (T1CE). The exclusion criteria were: (i) any tumor‐related treatment prior to MRI acquisition, including biopsy, radiotherapy, and chemotherapy; (ii) MRI artifacts related to motion or susceptibility. The patient selection flowchart is shown in Figure 1.

### 2.2 MRI Acquisitions

MRI studies were performed at six institutions using either a 3.0 T or a 1.5 T unit from different scanners, with various parameters to reflect real inter‐center heterogeneity. The brain tumor imaging protocol included T1WI, T2WI, FLAIR, and T1CE. The detailed acquisition parameters are provided in Table S1.

### 2.3 MRI Semantic Feature Extraction

All MRI data were anonymized and stored in Digital Imaging and Communications in Medicine format. Two senior radiologists (who had 7 and 10 years of neuroimaging experience, respectively) blinded to the histopathological diagnoses, independently assessed image features. Thirteen MRI semantic features were analyzed in this study, comprising 3 quantitative features and 10 qualitative features (detailed in Table S2). For intra‐observer consistency analysis, all cases were reassessed after a 2‐month washout period. Ultimately, discrepancies were resolved by a third radiologist with 15 years' neuroimaging experience for model development.

### 2.4 Model Construction

Patients from three centers (Centers A–C) were randomly allocated to the training and internal validation sets at a 7:3 ratio for model construction and evaluation, while cases from the other centers served as an independent external testing set for further model assessment.

#### 2.4.1 Quantitative Parametric Models

Quantitative features with statistically significant intergroup differences were firstly used to construct single‐parameter models to differentiate MB from EA. Subsequently, these features were integrated to establish a multivariable logistic regression model, hereafter denoted as the JOINT model.

#### 2.4.2 Mixed Parametric Models

Initially, clinical features (age and gender), as well as quantitative and qualitative MRI features were screened using least absolute shrinkage and selection operator (LASSO) regression and the Boruta algorithm in the training set. Features that were identified by both methods were retained for further analysis. Subsequently, Pearson correlation and variance inflation factor tests were conducted on the selected features to eliminate significant covariance or multicollinearity. Then, these features were used to construct five machine learning models, including adaptive boosting (AdaBoost), light gradient boosting machine (LightGBM), random forest (RF), multilayer perceptron (MLP), and support vector machine (SVM). Random search and manual fine‐tuning with 5‐fold cross‐validation were used to determine the optimal hyperparameters for each model. Finally, the diagnostic performance was evaluated by calculating area under the receiver operating characteristic curve (AUC) in both the internal validation and external testing datasets. Additionally, SHapley Additive exPlanations (SHAP) was employed to assess the importance of each feature in the best model.

### 2.5 Radiologist Assessment

To assess the clinical utility of the diagnostic model, the performance of radiologists' independent and model‐assisted assessments was evaluated. Firstly, the assessment was performed by three senior radiologists (who had 8, 9, and 10 years of neuroimaging experience, respectively) and three junior radiologists (who had 3, 4, and 5 years of neuroimaging experience, respectively) independently. All radiologists were blinded to histopathological results. Based on conventional MRI (T1WI, T2WI, and T1CE) and clinical data (age, gender, and onset), diagnoses were recorded on a 4‐point scale (1 = definite MB; 2 = likely MB; 3 = likely EA; 4 = definite EA). Subsequently, following a 1‐month washout period, these radiologists were provided with the binary output based on the optimal cutoff value along with its predicted probability, and then reassessed all cases.

### 2.6 Statistical Analysis

The normality of variables was assessed using the Shapiro–Wilk test. Quantitative variables were expressed as mean ± standard deviation for normally distributed data or as median with interquartile range (IQR) for non‐normally distributed data. Categorical variables were presented as numbers and percentages. For continuous variable comparisons between two groups, Student's *t*‐test was employed when data adhered to a Gaussian distribution; conversely, the Wilcoxon rank‐sum test was utilized for non‐normally distributed data. For categorical variables, the chi‐square test or Fisher's exact test was used. Intraclass correlation coefficient (ICC) was calculated to evaluate the intra‐ and inter‐observer agreement for quantitative features. Cohen's kappa analysis was performed to assess the intra‐ and inter‐observer agreement for qualitative features and radiologist diagnoses. Receiver operating characteristic (ROC) curves were constructed, and the optimal cut‐off value was identified using the maximal Youden index. The diagnostic efficacy of different analytical strategies was evaluated using accuracy, sensitivity, specificity, positive predictive value (PPV), and negative predictive value (NPV). The integrated discrimination improvement index (IDI) was used to assess the additive predictive value of the prediction model. Statistical analyses were performed using SPSS (version 27.0). Feature selection and IDI calculations were conducted using R software (version 4.3.2), and machine learning model construction was implemented in the Python environment (version 3.8.5). A two‐sided *p* < 0.05 was considered statistically significant.

## 3 Results

### 3.1 Patient Characteristics and MRI Semantic Features

The patient characteristics and MRI semantic features are shown in Table 1. In the training and internal validation sets, 182 patients were included, consisting of 114 MB cases (64 males; median age, 6.00 years; interquartile range [IQR], 4.00–9.00 years) and 68 EA cases (38 males; median age, 7.00 years; IQR, 4.00–10.50 years). In the external testing set, a total of 113 patients were enrolled, consisting of 70 MB cases (40 males; median age, 5.50 years; IQR, 3.00–9.00 years) and 43 EA cases (27 males; median age, 6.00 years; IQR, 4.00–9.00 years). No significant differences in age or gender were observed between MB and EA groups (*p >* 0.05).

**TABLE 1:** Patient characteristics and MRI semantic features.

	Training and internal validation set (*n* = 182)			External testing set (*n* = 113)		
	Medulloblastoma (*n* = 114)	Ependymoma (*n* = 68)	*p*	Medulloblastoma (*n* = 70)	Ependymoma (*n* = 43)	*p*
Age (years)^a^	6.00 (4.00, 9.00)	7.00 (4.00, 10.50)	0.491	5.50 (3.00, 9.00)	6.00 (4.00, 9.00)	0.771
Gender			0.973			0.553
Male	64 (56.14%)	38 (55.88%)		40 (57.14%)	27 (62.79%)	
Female	50 (43.86%)	30 (44.12%)		30 (42.86%)	16 (37.21%)	
LR/UL	0.98 ± 0.20	0.76 ± 0.22	**< 0.001**	1.02 ± 0.23	0.81 ± 0.20	**< 0.001**
AP/UL	0.88 ± 0.21	0.66 ± 0.23	**< 0.001**	0.90 ± 0.21	0.70 ± 0.22	**< 0.001**
LR/AP^a^	1.14 (1.02, 1.24)	1.17 (1.01, 1.31)	0.213	1.14 (1.02, 1.26)	1.14 (1.05, 1.24)	0.948
Tumor center off midline (> 1 cm)			**< 0.001**			**0.026**
Present	2 (1.75%)	14 (20.59%)		2 (2.86%)	7 (16.28%)	
Absent	112 (98.25%)	54 (79.41%)		68 (97.14%)	36 (83.72%)	
Extension into Luschka foramen			0.618			0.181
Present	68 (59.65%)	38 (55.88%)		40 (57.14%)	19 (44.19%)	
Absent	46 (40.35%)	30 (44.12%)		30 (42.86%)	24 (55.81%)	
Extension into spinal canal			**< 0.001**			**< 0.001**
Present	36 (31.58%)	47 (69.12%)		20 (28.57%)	32 (74.42%)	
Absent	78 (68.42%)	21 (30.88%)		50 (71.43%)	11 (25.58%)	
Peritumoral edema			**< 0.001**			**0.001**
Present	63 (55.26%)	15 (22.06%)		34 (48.57%)	8 (18.60%)	
Absent	51 (44.74%)	53 (77.94%)		36 (51.43%)	35 (81.40%)	
Cyst(s)			0.770			0.528
Present	105 (92.11%)	64 (94.12%)		62 (88.57%)	40 (93.02%)	
Absent	9 (7.89%)	4 (5.88%)		8 (11.43%)	3 (6.98%)	
Intratumoral hemorrhage			**< 0.001**			**< 0.001**
Present	3 (2.63%)	18 (26.47%)		4 (5.71%)	13 (30.23%)	
Absent	111 (97.37%)	50 (73.53%)		66 (94.29%)	30 (69.77%)	
Sea anemone sign			**< 0.001**			**< 0.001**
Present	44 (38.60%)	0 (0.00%)		28 (40.00%)	0 (0.00%)	
Absent	70 (61.40%)	68 (100.00%)		42 (60.00%)	43 (100.00%)	
Diffuse microcysts within enhancement			**< 0.001**			**< 0.001**
Present	3 (2.63%)	21 (30.88%)		2 (2.86%)	11 (25.58%)	
Absent	111 (97.37%)	47 (69.12%)		68 (97.14%)	32 (74.42%)	
Leptomeningeal involvement			**< 0.001**			**0.002**
Present	21 (18.42%)	1 (1.47%)		13 (18.57%)	0 (0.00%)	
Absent	93 (81.58%)	67 (98.53%)		57 (81.43%)	43 (100.00%)	
Spinal metastasis			**0.015**			**0.043**
Present	13 (11.40%)	1 (1.47%)		7 (10.00%)	0 (0.00%)	
Absent	101 (88.60%)	67 (98.53%)		63 (90.00%)	43 (100.00%)	

*Note:* Bold values indicate statistically significant differences (two‐tailed *P* < 0.05). Unless otherwise indicated, data are numbers of patients, and data in parentheses are percentages.

Abbreviations: AP/UL, ratio of the anterior–posterior diameter to the upper‐lower diameter; LR/AP, ratio of the left–right diameter to the anterior–posterior diameter; LR/UL, ratio of the left–right diameter to the upper‐lower diameter.

^a^ Data are the median, and data in parentheses are the interquartile range.

In the training and internal validation sets (*n* = 182), significant differences were observed in the quantitative and qualitative features between MB and EA. For quantitative features, MB exhibited a significantly higher ratio of the left–right diameter to the upper‐lower diameter (LR/UL) (MB, 0.98; EA, 0.76; *p* < 0.001) and ratio of the anterior–posterior diameter to the upper‐lower diameter (AP/UL) (MB, 0.88; EA, 0.66; *p* < 0.001) compared to EA. Regarding qualitative features, MB demonstrated a higher prevalence of peritumoral edema (MB, 55.26%; EA, 22.06%; *p* < 0.001), sea anemone sign (MB, 38.60%; EA, 0.00%; *p* < 0.001), leptomeningeal involvement (MB, 18.42%; EA, 1.47%; *p* < 0.001), and spinal metastasis (MB, 11.40%; EA, 1.47%; *p* = 0.015) compared to EA. In contrast, EA showed higher frequencies of tumor center off midline (EA, 20.59%; MB, 1.75%; *p* < 0.001), tumor extension into the spinal canal (EA, 69.12%; MB, 31.58%; *p* < 0.001), intratumoral hemorrhage (EA, 26.47%; MB, 2.63%; *p* < 0.001) and diffuse microcysts within enhancement (EA, 30.88%; MB, 2.63%; *p* < 0.001). Similar trends were observed in the external testing set (*n* = 113). Furthermore, the schematic diagrams of the sea anemone sign are illustrated in Figure S1 and its diagnostic performance for MB was assessed in three datasets, with accuracies of 0.594/0.667/0.628 and sensitivities/specificities of 0.350/1.000, 0.471/1.000, and 0.400/1.000 in the training, internal validation, and external testing sets, respectively.

**FIGURE 2:**
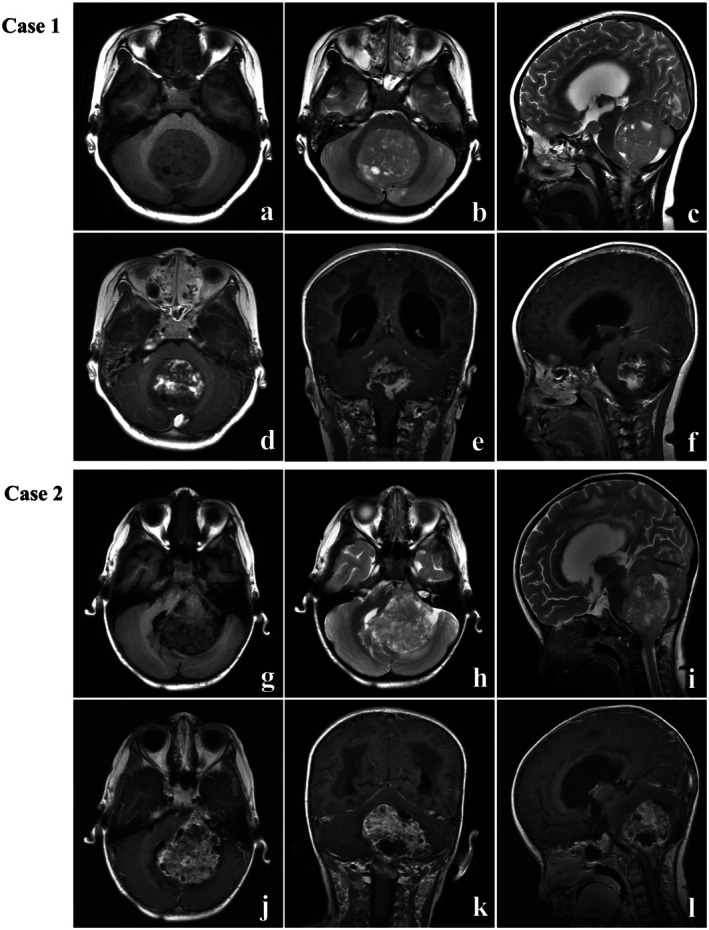
Representative cases of medulloblastoma and ependymoma. Case 1 (a–f), a 10‐year‐old girl with medulloblastoma in the fourth ventricle. The tumor showed hypointensity on T1WI (a), slight hyperintensity on axial T2WI (b) and sagittal T2WI (c). Triplanar T1CE (d–f) revealed marked heterogeneous enhancement with a sea anemone‐like pattern on coronal (e) and sagittal views (f). The quantitative parameters LR/UL and AP/UL values were 1.06 and 0.88, respectively. Case 2 (g–l), a 13‐year‐old boy with ependymoma in the fourth ventricle. The tumor exhibited hypointensity on T1WI (g), slight hyperintensity on axial T2WI (h) and sagittal T2WI (i). Triplanar T1CE (j–l) demonstrated moderate enhancement with diffuse microcystic areas, with multiple microcysts manifesting as focal T1 hypointensity and T2 hyperintensity. The quantitative parameters LR/UL and AP/UL values were 0.59 and 0.60, respectively. AP/UL, ratio of the anterior–posterior diameter to the upper‐lower diameter; LR/UL, ratio of the left–right diameter to the upper‐lower diameter; T1CE, contrast‐enhanced T1WI; T1WI, T1‐weighted imaging; T2WI, T2‐weighted imaging.

As shown in Table S3, all quantitative and qualitative features demonstrated excellent inter‐ and intra‐observer agreement (ICC, 0.908–0.975; *κ*, 0.820–1.000). Representative cases of MB and EA are shown in Figure 2.

### 3.2 Performance of Quantitative and Mixed Parametric Models

Table 2 presents the diagnostic performance of quantitative and mixed parametric models for differentiating MB from EA. For quantitative parametric models, the AUC values of the AP/UL model in the training, internal validation, and external testing sets were 0.788, 0.776, and 0.773, respectively, while those of the LR/UL model were 0.770, 0.782, and 0.779, respectively. The corresponding ACC values were 0.797, 0.759, and 0.752 for the AP/UL model, and 0.773, 0.778, and 0.735 for the LR/UL model. The JOINT model did not show significant performance improvement, achieving AUC/ACC values of 0.790/0.812 (training set), 0.804/0.778 (internal validation set), and 0.796/0.761 (external testing set).

**TABLE 2:** Performance of quantitative and mixed parametric models for differentiating medulloblastoma from ependymoma.

Models	AUC (95% CI)	Accuracy	Sensitivity	Specificity	PPV	NPV	Precision	Recall	F1
**Quantitative parametric models**									
LR/UL									
Training set	0.770 (0.681–0.859)	0.773	0.838	0.667	0.807	0.711	0.807	0.838	0.822
Internal validation set	0.782 (0.635–0.930)	0.778	0.882	0.600	0.789	0.750	0.789	0.882	0.833
External testing set	0.779 (0.686–0.872)	0.735	0.871	0.512	0.744	0.710	0.744	0.871	0.803
AP/UL									
Training set	0.788 (0.697–0.880)	0.797	0.862	0.688	0.821	0.750	0.821	0.862	0.841
Internal validation set	0.776 (0.635–0.918)	0.759	0.882	0.550	0.769	0.733	0.769	0.882	0.822
External testing set	0.773 (0.675–0.870)	0.752	0.843	0.605	0.776	0.703	0.776	0.843	0.808
JOINT									
Training set	0.790 (0.698–0.882)	0.812	0.900	0.667	0.818	0.800	0.818	0.900	0.857
Internal validation set	0.804 (0.670–0.939)	0.778	0.912	0.550	0.775	0.786	0.775	0.912	0.838
External testing set	0.796 (0.701–0.890)	0.761	0.871	0.581	0.772	0.735	0.772	0.871	0.819
**Mixed parametric models**									
AdaBoost									
Training set	0.981 (0.964–0.999)	0.922	0.962	0.854	0.917	0.932	0.917	0.962	0.939
Internal validation set	0.932 (0.863–1.000)	0.870	0.912	0.800	0.886	0.842	0.886	0.912	0.899
External testing set	0.923 (0.874–0.973)	0.814	0.957	0.581	0.788	0.893	0.788	0.957	0.865
LightGBM									
Training set	0.886 (0.830–0.942)	0.812	0.812	0.812	0.878	0.722	0.878	0.812	0.844
Internal validation set	0.838 (0.725–0.950)	0.741	0.794	0.650	0.794	0.650	0.794	0.794	0.794
External testing set	0.854 (0.784–0.923)	0.752	0.771	0.721	0.818	0.660	0.818	0.771	0.794
RF									
Training set	0.999 (0.998–1.000)	0.992	1.000	0.979	0.988	1.000	0.988	1.000	0.994
Internal validation set	0.926 (0.862–0.991)	0.778	0.882	0.600	0.789	0.750	0.789	0.882	0.833
External testing set	0.871 (0.798–0.943)	0.796	0.886	0.651	0.805	0.778	0.805	0.886	0.844
MLP									
Training set	0.937 (0.894–0.981)	0.883	0.938	0.792	0.882	0.884	0.882	0.938	0.909
Internal validation set	0.925 (0.843–1.000)	0.870	0.912	0.800	0.886	0.842	0.886	0.912	0.899
External testing set	0.911 (0.851–0.969)	0.814	0.886	0.698	0.827	0.789	0.827	0.886	0.855
SVM									
Training set	0.946 (0.899–0.993)	0.906	0.888	0.938	0.959	0.833	0.959	0.888	0.922
Internal validation set	0.921 (0.819–1.000)	0.907	0.912	0.900	0.939	0.857	0.939	0.912	0.925
External testing set	0.915 (0.852–0.979)	0.832	0.843	0.814	0.881	0.761	0.881	0.843	0.861

Abbreviations: AdaBoost, adaptive boosting; AP/UL, the ratio of the anterior–posterior diameter to the upper‐lower diameter; AUC, area under the curve; CI, confidence interval; JOINT, logistic regression model constructed using LR/UL and AP/UL; LightGBM, light gradient boosting machine; LR/UL, the ratio of the left–right diameter to the upper‐lower diameter; MLP, multilayer perceptron; NPV, negative predictive value; PPV, positive predictive value; RF, random forest; SVM, support vector machine.

**FIGURE 3:**
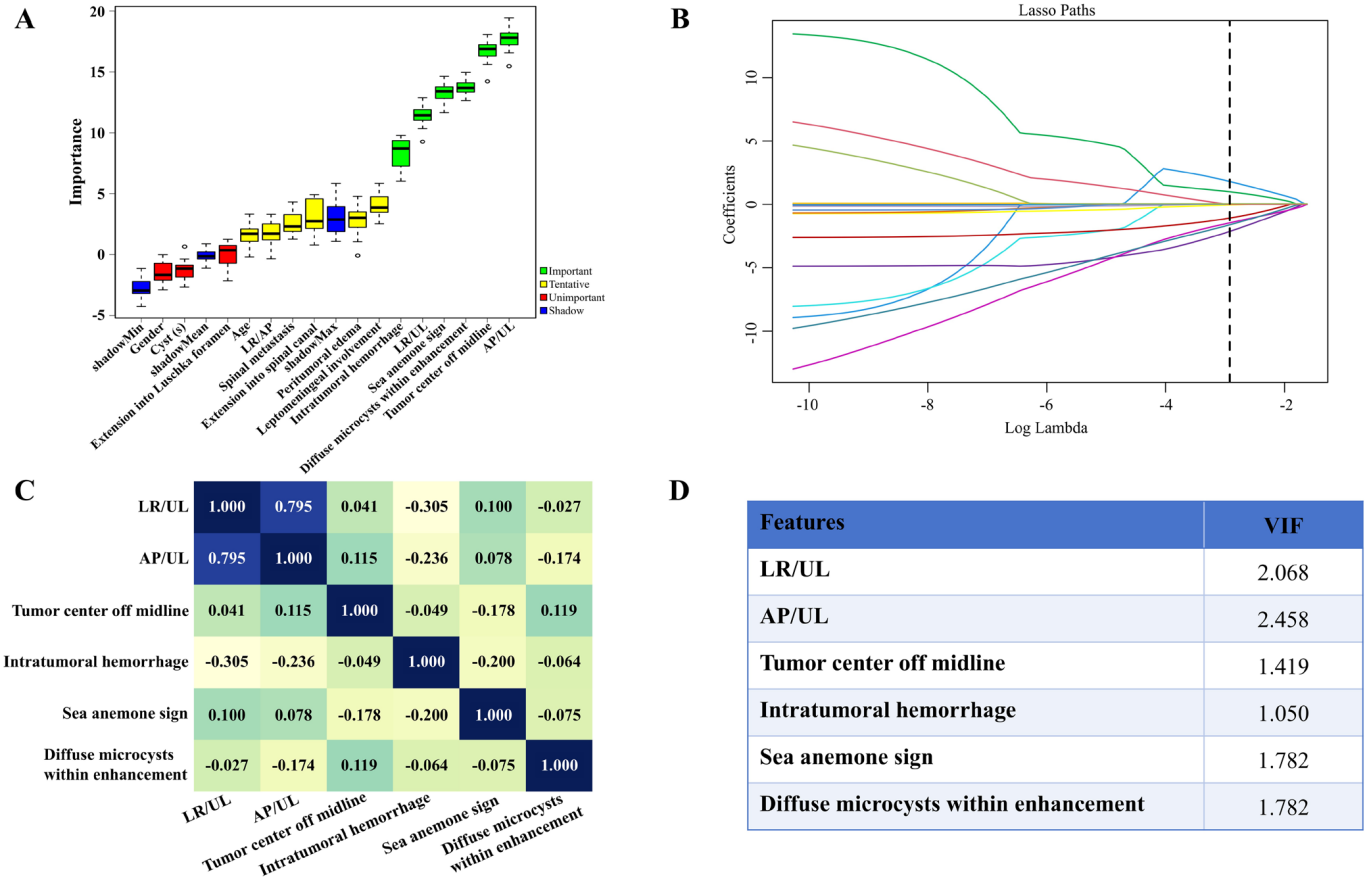
Feature selection and evaluation. Clinical features and MRI features were screened using the Boruta (A) and LASSO regression (B). Pearson correlation (C) and variance inflation factor tests (D) were conducted on the intersection of features. LASSO, least absolute shrinkage and selection operator; AP/UL, ratio of the anterior–posterior diameter to the upper‐lower diameter; LR/UL, ratio of the left–right diameter to the upper‐lower diameter.

As illustrated in Figure 3, six important features were selected by LASSO regression and Boruta algorithm intersection, including LR/UL, AP/UL, tumor center deviation off the midline, intratumoral hemorrhage, sea anemone sign, and diffuse microcysts within enhancement (Figure 3A,B). Pearson correlation analysis (Figure 3C) and variance inflation factor (Figure 3D) confirmed the absence of strong correlations or multicollinearity among these features.

Based on these six features, five models (AdaBoost, LightGBM, RF, MLP, and SVM) were subsequently developed. As shown in Table 2, the SVM model demonstrated superior diagnostic performance with optimal generalization capacity, achieving AUCs of 0.946/0.921/0.915 and accuracies of 0.906/0.907/0.832 across training/internal validation/external testing sets. IDI analysis (Tables S4 and S5) demonstrated significantly improved diagnostic performance of the SVM model compared with the JOINT model and all other machine learning models except the RF model, with IDI values of 0.197–0.476 (training), 0.145–0.436 (internal validation), and 0.168–0.399 (external testing). When comparing the SVM model with the RF model, the IDI was −0.228 (*p* < 0.001) in the training set and 0.108 (*p* = 0.046) in the external testing set, with no statistical significance in the internal validation set (IDI = 0.013, *p* = 0.783). As presented in Figure S2A–C, calibration curves demonstrated good calibration for the SVM model. Decision curve analysis demonstrated superior clinical net benefit of the SVM model compared to the JOINT model (Figure S2D–F). To assess the relative influence of model variables, SHAP analysis was utilized in the SVM model. As shown in Figure S3, AP/UL contributed as the strongest positive predictor, while diffuse microcysts within enhancement exhibited the most significant negative impact in SVM predictions.

### 3.3 Performance of Radiologists' Independent and Model‐Assisted Assessment

**FIGURE 4:**
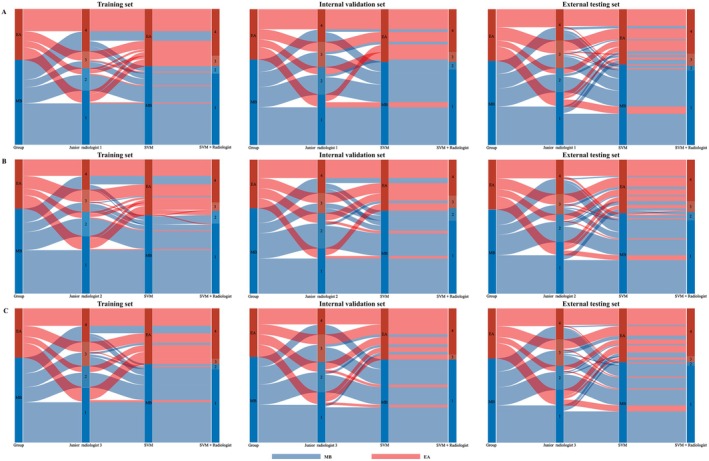
Sankey diagram of diagnostic transitions from independent to model‐assisted assessment for junior radiologists. The width of each flow represents the number of cases transitioning between diagnostic states. SVM, support vector machine.

**FIGURE 5:**
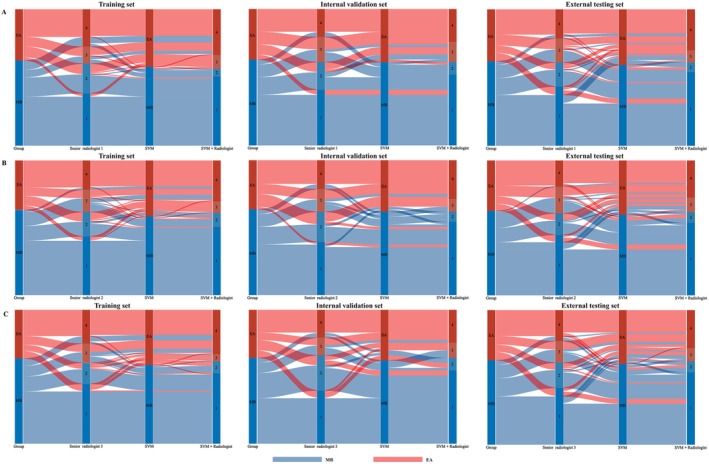
Sankey diagram of diagnostic transitions from independent to model‐assisted assessment for senior radiologists. The width of each flow represents the number of cases transitioning between diagnostic states. SVM, support vector machine.

Tables 3 and 4 present the performance of junior and senior radiologists' independent and model‐assisted assessment, respectively. In independent assessment, senior radiologists exhibited superior diagnostic performance compared with junior radiologists, with AUCs of 0.812–0.867, 0.793–0.846, and 0.801–0.838 and accuracies of 0.766–0.781, 0.759, 0.752–0.770 in the training, internal validation, and external testing sets, respectively. In contrast, junior radiologists achieved lower AUCs of 0.632–0.667, 0.652–0.671, 0.631–0.656 and accuracies of 0.625–0.680, 0.630–0.667, 0.628–0.664 in the respective sets. When assisted by the SVM model, diagnostic performance improved for both senior and junior radiologists in the training, internal validation, and external testing sets. In the external testing set, the AUCs of junior radiologists increased to 0.849–0.893 and the accuracies to 0.832–0.858 while the AUCs of senior radiologists increased to 0.893–0.913 and the accuracies to 0.850–0.867. The diagnostic shifts from independent to model‐assisted assessment for junior and senior radiologists are illustrated in the Sankey diagrams in Figures 4 and 5, respectively. Model assistance resulted in the rectification of misdiagnosed cases among both junior and senior radiologists across the training, internal validation, and external testing sets, accompanied by an increase in their diagnostic confidence.

**TABLE 3:** Performance comparison of junior radiologists' independent and model‐assisted assessment in differentiating medulloblastoma from ependymoma.

Models	AUC (95% CI)	Accuracy^a^	Accuracy^b^	Sensitivity	Specificity	PPV	NPV	Precision	Recall	F1
**Trai**n**ing set**										
Radiologist										
Junior radiologist 1	0.654 (0.560–0.748)	0.656	0.469	0.675	0.625	0.750	0.536	0.750	0.675	0.711
Junior radiologist 2	0.667 (0.573–0.762)	0.680	0.453	0.725	0.604	0.753	0.569	0.753	0.725	0.739
Junior radiologist 3	0.632 (0.535–0.729)	0.625	0.438	0.650	0.583	0.722	0.500	0.722	0.650	0.684
Radiologist + SVM										
Junior radiologist 1	0.908 (0.855–0.960)	0.906	0.781	0.888	0.938	0.959	0.833	0.959	0.888	0.922
Junior radiologist 2	0.891 (0.834–0.949)	0.875	0.750	0.888	0.854	0.910	0.820	0.910	0.888	0.899
Junior radiologist 3	0.914 (0.865–0.964)	0.906	0.836	0.888	0.938	0.959	0.833	0.959	0.888	0.922
**Internal validation set**										
Radiologist										
Junior radiologist 1	0.662 (0.512–0.813)	0.648	0.426	0.676	0.600	0.742	0.522	0.742	0.676	0.708
Junior radiologist 2	0.671 (0.523–0.818)	0.667	0.407	0.706	0.600	0.750	0.545	0.750	0.706	0.727
Junior radiologist 3	0.652 (0.501–0.804)	0.630	0.407	0.676	0.550	0.719	0.500	0.719	0.676	0.697
Radiologist + SVM										
Junior radiologist 1	0.908 (0.820–0.996)	0.907	0.796	0.912	0.900	0.939	0.857	0.939	0.912	0.925
Junior radiologist 2	0.899 (0.807–0.990)	0.889	0.741	0.912	0.850	0.912	0.850	0.912	0.912	0.912
Junior radiologist 3	0.901 (0.816–0.987)	0.907	0.870	0.912	0.900	0.939	0.857	0.939	0.912	0.925
**External testing set**										
Radiologist										
Junior radiologist 1	0.645 (0.541–0.748)	0.646	0.416	0.671	0.605	0.734	0.531	0.734	0.671	0.701
Junior radiologist 2	0.656 (0.555–0.758)	0.664	0.434	0.700	0.605	0.742	0.553	0.742	0.700	0.721
Junior radiologist 3	0.631 (0.526–0.736)	0.628	0.416	0.657	0.581	0.719	0.510	0.719	0.657	0.687
Radiologist + SVM										
Junior radiologist 1	0.875 (0.807–0.943)	0.841	0.779	0.843	0.837	0.894	0.766	0.894	0.843	0.868
Junior radiologist 2	0.893 (0.829–0.956)	0.858	0.779	0.871	0.837	0.897	0.800	0.897	0.871	0.884
Junior radiologist 3	0.849 (0.778–0.921)	0.832	0.796	0.843	0.814	0.881	0.761	0.881	0.843	0.861

Abbreviations: AUC, area the under curve; CI, confidence interval; NPV, negative predictive value; PPV, positive predictive value; SVM, support vector machine.

^a^ Accuracy = Number of cases with (possible diagnosis (scale 2 and 3) and definite diagnosis (scale 1 and 4))/number of cases.

^b^ Accuracy = Number of cases with definite diagnosis (scale 1 and 4)/number of cases.

**TABLE 4:** Performance comparison of senior radiologists' independent and model‐assisted assessment in differentiating medulloblastoma from ependymoma.

Models	AUC (95% CI)	Accuracy^a^	Accuracy^b^	Sensitivity	Specificity	PPV	NPV	Precision	Recall	F1
**Training set**										
Radiologist										
Senior radiologist 1	0.831 (0.761–0.900)	0.773	0.563	0.800	0.729	0.831	0.686	0.831	0.800	0.815
Senior radiologist 2	0.867 (0.803–0.931)	0.781	0.609	0.813	0.729	0.833	0.700	0.833	0.813	0.823
Senior radiologist 3	0.812 (0.735–0.889)	0.766	0.594	0.788	0.729	0.829	0.673	0.829	0.788	0.808
Radiologist + SVM										
Senior radiologist 1	0.915 (0.865–0.964)	0.906	0.766	0.875	0.958	0.972	0.821	0.972	0.875	0.921
Senior radiologist 2	0.935 (0.890–0.979)	0.914	0.758	0.913	0.917	0.948	0.863	0.948	0.913	0.930
Senior radiologist 3	0.909 (0.858–0.960)	0.867	0.781	0.875	0.854	0.909	0.804	0.909	0.875	0.892
**Internal validation set**										
Radiologist										
Senior radiologist 1	0.821 (0.707–0.936)	0.759	0.537	0.794	0.700	0.818	0.667	0.818	0.794	0.806
Senior radiologist 2	0.846 (0.744–0.949)	0.759	0.556	0.794	0.700	0.818	0.667	0.818	0.794	0.806
Senior radiologist 3	0.793 (0.665–0.920)	0.759	0.519	0.824	0.650	0.800	0.684	0.800	0.824	0.812
Radiologist + SVM										
Senior radiologist 1	0.918 (0.832–1.000)	0.870	0.722	0.882	0.850	0.909	0.810	0.909	0.882	0.896
Senior radiologist 2	0.920 (0.834–1.000)	0.907	0.778	0.912	0.900	0.939	0.857	0.939	0.912	0.925
Senior radiologist 3	0.903 (0.814–0.992)	0.852	0.741	0.882	0.800	0.882	0.800	0.882	0.882	0.882
**External testing set**										
Radiologist										
Senior radiologist 1	0.808 (0.725–0.892)	0.761	0.558	0.800	0.698	0.812	0.682	0.812	0.800	0.806
Senior radiologist 2	0.838 (0.761–0.916)	0.770	0.584	0.800	0.721	0.824	0.689	0.824	0.800	0.812
Senior radiologist 3	0.801 (0.716–0.887)	0.752	0.558	0.786	0.698	0.809	0.667	0.809	0.786	0.797
Radiologist + SVM										
Senior radiologist 1	0.898 (0.836–0.961)	0.867	0.770	0.886	0.837	0.899	0.818	0.899	0.886	0.892
Senior radiologist 2	0.913 (0.856–0.971)	0.867	0.761	0.886	0.837	0.899	0.818	0.899	0.886	0.892
Senior radiologist 3	0.893 (0.829–0.956)	0.850	0.752	0.871	0.814	0.884	0.795	0.884	0.871	0.878

Abbreviations: AUC, area under the curve; CI, confidence interval; NPV, negative predictive value; PPV, positive predictive value; SVM, support vector machine.

^a^ Accuracy = Number of cases with (possible diagnosis (scale 2 and 3) and definite diagnosis (scale 1 and 4))/number of cases.

^b^ Accuracy = Number of cases with definite diagnosis (scale 1 and 4)/number of cases.

As shown in Table S6, both senior radiologists and junior radiologists exhibited good inter‐observer agreement (junior radiologists, 0.618; senior radiologists, 0.694) in independent assessment, while assisted by the SVM model, both groups showed excellent inter‐observer agreement (junior radiologists, 0.802; senior radiologists, 0.802).

## 4 Discussion

In this multi‐center study, we identified six key features for differentiating pediatric MB and EA in the fourth ventricle, including LR/UL, AP/UL, tumor center off midline, intratumoral hemorrhage, sea anemone sign, and diffuse microcysts within enhancement. Mixed parametric models outperformed quantitative parametric models, with the SVM model exhibiting optimal discriminative power. Furthermore, the diagnostic performance of both senior and junior radiologists was improved when assisted with the SVM model.

In this study, the quantitative parameters LR/UL and AP/UL showed statistically significant differences between MB and EA with minimal overlap, indicating potential clinical utility in differentiating these conditions. To our knowledge, this study represents the first attempt to utilize diameter ratio analysis for differentiating MB from EA. Our quantitative analysis revealed significantly elevated LR/UL and AP/UL ratios in MB relative to EA. Histopathologically, MB is characterized by densely packed small tumor cells with scant myxoid matrix, conferring firmer, friable tissue consistency, limited deformability, and predominantly expansile growth. In contrast, EA demonstrates moderate cellular density with frequent myxoid matrix, imparting a softer consistency, greater deformability, and conformational growth along ventricular spaces [22, 23]. These histopathological characteristics underlie the morphological differences wherein MB demonstrates reduced UL dimension with greater LR and AP expansion compared to EA at matched volumes. Consistent with our findings, Zhang et al. [13]. reported that significantly higher sphericity was observed in MB compared to EA, indicating its more spherical morphology. However, sphericity extraction necessitates whole‐tumor delineation—a labor‐intensive process that impedes clinical adoption. In contrast, diameter ratio measurements offer practical advantages through their simplicity, rapid assessment capability, and seamless integration into routine clinical workflows.

Qualitatively, two enhancement‐based imaging signs were identified as critical discriminators between MB and EA. First, the sea anemone sign was highly specific to the MB group and was not observed in any EA cases. While potentially reflecting heterogeneous vascular distribution, its underlying pathophysiological basis requires further investigation [17]. Second, diffuse microcysts within enhancement were typically observed in EA, likely associated with EA's inherent propensity for myxoid or cystic degeneration [24]. Additionally, tumor center off midline was more common in EA, attributed to its “plastic” nature enabling it to spread more extensively than MB [25]. Notably, intratumoral hemorrhage occurred in only 2.63% of MB cases in our study, which was lower than the 5%–15% in the literature [26]. This discrepancy may be attributed to the fact that our study focused on children under 14 years old with MB and EA in the fourth ventricle.

Our study found that the SVM model achieved optimal diagnostic efficacy in differentiating MB from EA. Model performance depends critically on feature dimensionality and complexity. Due to the relatively simple feature space and small sample size, using only six features better suited SVM. In such cases, complex models risk overfitting or undertraining, thereby reducing efficacy [27, 28, 29, 30].

This study has several limitations. First, although data were collected from multiple centers, the sample size remains relatively limited, and the retrospective design may introduce inherent biases. Second, the pathological basis of key imaging features could not be directly validated due to the lack of histopathological specimens. Future studies incorporating histopathological correlation and detailed anatomical analysis may further elucidate these imaging findings. Third, molecular subgroup analysis was not available and should be explored in future research.

## 5 Conclusion

MB exhibits higher LR/UL and AP/UL ratios than EA, with the sea anemone sign serving as a highly specific enhancement feature. We developed SVM models based on conventional imaging features, which demonstrated excellent diagnostic performance in preoperative differentiation of MB from EA, potentially enhancing radiologists' diagnostic accuracy in clinical practice.

## Funding

This study received financial support from the National Natural Science Foundation of China (No. 82371936 to YBX), the 7T MRI Precision Neurology Platform of Shaanxi Province (No. 2025PT‐08 to WW), and the Key Core Technique Program of Shaanxi Province (No. 2024SF2‐GJHX‐71 to WW).

## Ethics Statement

This study was approved by the institutional review board of Tangdu Hospital (K‐HG‐202507‐05). Due to the retrospective nature of this study, the requirement for informed consent was waived.

## Conflicts of Interest

The authors declare no conflicts of interest.

## Supporting information


**Table S1:** Detailed information of MRI parameters.
**Table S2:** MRI semantic features characterization.
**Table S3:** Consistency analyses of MRI features.
**Table S4:** Integrated discrimination improvement (IDI) of mixed parametric models relative to the JOINT model.
**Table S5:** Integrated discrimination improvement (IDI) of SVM model relative to the other mixed parametric models.
**Table S6:** Consistency analyses of diagnostic results with and without model assistance for junior and senior radiologists.
**Figure S1:** Schematic diagrams of the sea anemone sign. (A) Schematic diagram (left) and representative case (right) of the sea anemone sign with a central enhancing trunk. (B) Schematic diagram (left) and representative case (right) of the sea anemone sign without a central enhancing trunk.
**Figure S2:** Calibration (A‐C) and decision curve analysis curves (D‐F) of SVM Model in the training, internal validation and external testing sets. Abbreviations: SVM = support vector machine.
**Figure S3:** SHAP value of features for SVM model prediction. Abbreviations: LR/UL = the ratio of the left–right diameter to the upper‐lower diameter, AP/UL = the ratio of the anterior–posterior diameter to the upper‐lower diameter.

## Data Availability

The data that support the findings of this study are available from the corresponding author upon reasonable request.
