# Supplementary material for: Conventional MRI‐Based Semantic Features for Differentiation of Pediatric Medulloblastoma and Ependymoma in the Fourth Ventricle: Insights From a Multi‐Center Retrospective Study
Source: CNS Neurosci Ther. 2026 Jun 24;32(6):e70999. doi: 10.1002/cns.70999 (PMC13292730; doi:10.1002/cns.70999)
Supplement: Supplementary file 1 — Table S1: Detailed information of MRI parameters. Table S2: MRI semantic features characterization. Table S3: Consistency analyses of MRI features. Table S4: Integrated discrimination improvement (IDI) of mixed parametric models relative to the JOINT model. Table S5: Integrated discrimination improvement (IDI) of SVM model relative to the other mixed parametric models. Table S6: Consistency analyses of diagnostic results with and without model assistance for junior and senior radiologists. Figure S1: Schematic diagrams of the sea anemone sign. (A) Schematic diagram (left) and representative case (right) of the sea anemone sign with a central enhancing trunk. (B) Schematic diagram (left) and representative case (right) of the sea anemone sign without a central enhancing trunk. Figure S2: Calibration (A‐C) and decision curve analysis curves (D‐F) of SVM Model in the training, internal validation and external testing sets. Abbreviations: SVM = support vector machine. Figure S3: SHAP value of features for SVM model prediction. Abbreviations: LR/UL = the ratio of the left–right diameter to the upper‐lower diameter, AP/UL = the ratio of the anterior–posterior diameter to the upper‐lower diameter. [file CNS-32-e70999-s001.docx]

**Table S1. Detailed information of MRI parameters.**

|  | **TR (ms)** | **TE (ms)** | **Thickness (mm)** | **Spacing (mm)** |
| --- | --- | --- | --- | --- |
| **Tangdu Hospital** **(Center A)**  **(GE Discovery 750 3.0T/Siemens MAGNETOM Aera 1.5T)** |  |  |  |  |
| **T1WI** | 450/1750 | 10/24 | 5/5.5 | 1/1.5 |
| **T2WI** | 4247/4300 | 93/109 | 5/5.5 | 1/1.5 |
| **T1CE** | 450/1750 | 8.9/24 | 5/5.5 | 1/1.5 |
| **Xi’an People’s Hospital (Center B)**  **(Siemens MAGNETOM Prisma 3.0T/Siemens MAGNETOM Aera 1.5T)** |  |  |  |  |
| **T1WI** | 2000/2000 | 18/19 | 5.5/6 | 1/1.5 |
| **T2WI** | 4500/5000 | 95/96 | 5.5/6 | 1/1.5 |
| **T1CE** | 559/2040 | 8.9/9 | 5.5/6 | 1/1.5 |
| **Xi’an Children’s Hospital (Center C)**  **(****Philips Achieva 3.0 TX)** |  |  |  |  |
| **T1WI** | 2977 | 31 | 5 | 2 |
| **T2WI** | 5968 | 111 | 5 | 2 |
| **T1CE** | 1450 | 24 | 5 | 2 |
| **Qinghai Provincial People’s Hospital (Center D)**  **(Siemens MAGNETOM Skyra 3.0T)** |  |  |  |  |
| **T1WI** | 2100 | 9 | 5 | 1 |
| **T2WI** | 5790 | 142 | 5 | 1 |
| **T1CE** | 2260 | 9 | 5 | 1 |
| **Lanzhou University Second Hospital (Center E)**  **(GE SIGNA Premier 3.0T)** |  |  |  |  |
| **T1WI** | 1772 | 26 | 5 | 1 |
| **T2WI** | 5444 | 82.5 | 5 | 1 |
| **T1CE** | 7.2 | 3 | 1 | 1 |
| **Chengdu First People’s Hospital (Center F)**  **(Philips Achieva 3.0 TX)** |  |  |  |  |
| **T1WI** | 2000 | 20 | 6.5 | 1 |
| **T2WI** | 3000 | 80 | 6.5 | 1 |
| **T1CE** | 3.5 | 1.73 | 2.4 | -1.2 |

**Abbreviations:** T1WI = T1-weighted imaging, T2WI = T2-weighted imaging, T1CE = contrast-enhanced T1-weighted imaging, TR = repetition time, TE = echo time.

**Table S2. MRI semantic features characterization.**

| **MRI features** | **Categories** | **Description** |
| --- | --- | --- |
| **LR/UL** | Quantitative parametric | The ratio of the maximum left-right diameter (measured on the axial plane) to the maximum upper-lower diameter (measured on the sagittal plane), with tumor boundaries delineated on jointly interpreted multiplanar T1WI, T2WI, and T1CE |
| **AP/UL** | Quantitative parametric | The ratio of the maximum anterior-posterior diameter (measured on the sagittal plane) to the maximum upper-lower diameter (measured on the sagittal plane), with tumor boundaries delineated on jointly interpreted multiplanar T1WI, T2WI, and T1CE |
| **LR/AP** | Quantitative parametric | The ratio of the maximum left-right diameter (measured on the axial plane) to the maximum anterior-posterior diameter (measured on the sagittal plane), with tumor boundaries delineated on jointly interpreted multiplanar T1WI, T2WI, and T1CE |
| **Tumor center off midline** | Present / Absent | Location of center of tumor in horizontal axis in relation to midline. Centre of tumor deviation from the midline > 1cm is considered as present. |
| **Extension into Luschka foramen** | Present / Absent | Relationship of tumor with the foramen of Luschka |
| **Extension into spinal canal** | Present / Absent | Relationship of tumor with the spinal canal |
| **Peritumoral edema** | Present / Absent | Patchy hyperintense on T2WI and hypointense on T1WI beyond the tumor edge |
| **Cyst (s)** | Present / Absent | Cystic components within the tumor |
| **Intratumoral hemorrhage** | Present / Absent | Hyperintensity on T1WI and hypointensity on T2WI within the tumor |
| **Sea anemone sign** | Present / Absent | Intratumoral focal radial enhancement with or without a central enhancing trunk, mimicking the morphological appearance of a sea anemone, predominantly on coronal and sagittal T1CE |
| **Diffuse microcysts within enhancement** | Present / Absent | The entire tumor shows marked enhancement, with more than 10 microcyst-like (≤ 5mm) non-enhancing regions distributed across multiple areas of the lesion on T1CE |
| **Leptomeningeal involvement** | Present / Absent | Leptomeningeal nodules on T2WI, or focal leptomeningeal enhancement on T1CE |
| **Spinal metastasis** | Present / Absent | Spinal nodules on T2WI, or focal spinal enhancement on T1CE |

**Abbreviations:** LR/UL = the ratio of the left-right diameter to the upper-lower diameter, AP/UL = the ratio of the anterior-posterior diameter to the upper-lower diameter, LR/AP = the ratio of the left-right diameter to the anterior-posterior diameter, T1WI = T1-weighted imaging, T2WI = T2-weighted imaging, T1CE = contrast-enhanced T1-weighted imaging

**Table S3. Consistency analyses of MRI features.**

| **MRI Features** | **Inter-observer agreement** | **Intra-observer agreement** |
| --- | --- | --- |
| **LR/UL** | 0.910 (0.750 - 0.990) | 0.972 (0.953 - 0.986) |
| **AP/UL** | 0.908 (0.782 - 0.982) | 0.975 (0.942 - 0.992) |
| **LR/AP** | 0.917 (0.855 - 0.953) | 0.942 (0.868 - 0.983) |
| **Tumor center off midline** | 0.834 (0.669 - 0.965) | 0.965 (0.880 - 1.000) |
| **Extension into Luschka foramen** | 0.922 (0.857 - 0.967) | 0.977 (0.942 - 1.000) |
| **Extension into spinal canal** | 0.901 (0.834 - 0.956) | 0.978 (0.944 - 1.000) |
| **Peritumoral edema** | 0.910 (0.842 - 0.966) | 0.966 (0.921 - 1.000) |
| **Cyst (s)** | 0.960 (0.852 - 1.000) | 0.963 (0.871 - 1.000) |
| **Intratumoral hemorrhage** | 0.914 (0.800 - 1.000) | 1.000 (1.000 - 1.000) |
| **Sea anemone sign** | 0.820 (0.709 - 0.911) | 0.882 (0.787 - 0.955) |
| **Diffuse microcysts within enhancement** | 0.865 (0.742 - 0.958) | 0.910 (0.808 - 0.980) |
| **Leptomeningeal involvement** | 0.924 (0.822- 1.000) | 0.975 (0.910 - 1.000) |
| **Spinal metastasis** | 0.960 (0.861- 1.000) | 1.000 (1.000 - 1.000) |

Note. — Data in parentheses are 95% confidence intervals. All quantitative parameters, including LR/UL, AP/UL, and LR/AP, were assessed using intraclass correlation coefficient, while all qualitative parameters were evaluated using Kappa analysis for consistency analyses. **Abbreviations:** LR/UL = ratio of the left-right diameter to the upper-lower diameter, AP/UL = ratio of the anterior-posterior diameter to the upper-lower diameter, LR/AP = ratio of the left-right diameter to the anterior-posterior diameter.

**Table S4. Integrated discrimination improvement (IDI) of mixed parametric models relative to the JOINT model.**

| **Models** | **IDI (95%CI)** | ***P* value** |
| --- | --- | --- |
| **AdaBoost vs JOINT** |  |  |
| Training set | 0.177 (0.092 - 0.263) | < 0.001 |
| Internal validation set | 0.172 (0.007 - 0.338) | 0.041 |
| External testing set | 0.143 (0.042 - 0.244) | 0.006 |
| **LightGBM vs JOINT** |  |  |
| Training set | 0.020 (-0.110 - -0.006) | 0.029 |
| Internal validation set | -0.004 (-0.069 - 0.061) | 0.915 |
| External testing set | -0.003 (-0.054 - 0.047) | 0.905 |
| **RF vs JOINT** |  |  |
| Training set | 0.603 (0.534 - 0.671) | < 0.001 |
| Internal validation set | 0.305 (0.156 - 0.453) | < 0.001 |
| External testing set | 0.203 (0.076 - 0.331) | 0.002 |
| **MLP vs JOINT** |  |  |
| Training set | -0.102 (-0.178 - -0.026) | 0.009 |
| Internal validation set | -0.118 (-0.244 - 0.008) | 0.066 |
| External testing set | -0.088 (-0.160 - -0.016) | 0.017 |
| **SVM vs JOINT** |  |  |
| Training set | 0.375 (0.279 - 0.470) | < 0.001 |
| Internal validation set | 0.318 (0.166 - 0.470) | < 0.001 |
| External testing set | 0.311 (0.207 - 0.415) | < 0.001 |

**Abbreviations:** CI = confidence interval, JOINT = logistic regression model constructed using LR/UL and AP/UL, AdaBoost = adaptive boosting, LightGBM = light gradient boosting machine, RF = random forest, MLP = multilayer perceptron, SVM = support vector machine.

**Table S5. Integrated discrimination improvement (IDI) of SVM model relative to the other mixed parametric models.**

| **Models** | **IDI (95%CI)** | ***P* value** |
| --- | --- | --- |
| **SVM vs AdaBoost** |  |  |
| Training set | 0.197 (0.124 - 0.270) | < 0.001 |
| Internal validation set | 0.145 (0.047 - 0.244) | 0.004 |
| External testing set | 0.168 (0.079 - 0.257) | < 0.001 |
| **SVM vs LightGBM** |  |  |
| Training set | 0.354 (0.268 - 0.440) | < 0.001 |
| Internal validation set | 0.321 (0.168 - 0.474) | < 0.001 |
| External testing set | 0.314 (0.219 - 0.408) | < 0.001 |
| **SVM vs RF** |  |  |
| Training set | -0.228 (-0.298 - -0.159) | < 0.001 |
| Internal validation set | 0.013 (-0.079 - 0.105) | 0.783 |
| External testing set | 0.108 (0.002 - 0.213) | 0.046 |
| **SVM vs MLP** |  |  |
| Training set | 0.476 (0.417 - 0.536) | < 0.001 |
| Internal validation set | 0.436 (0.339 - 0.532) | < 0.001 |
| External testing set | 0.399 (0.327 - 0.471) | < 0.001 |

**Abbreviations:** CI = confidence interval, AdaBoost = adaptive boosting, LightGBM = light gradient boosting machine, RF = random forest, MLP = multilayer perceptron, SVM = support vector machine.

**Table S****6.** **Consistency analyses of diagnostic results with and without model assistance for junior and senior radiologists.**

| **Characteristic** | ***κ* value** |  |
| --- | --- | --- |
| **Radiologist** |  | |
| Junior radiologists | 0.618 (0.557 - 0.678) | |
| Senior radiologists | 0.694 (0.634 - 0.755) | |
| **Radiologist + SVM** |  | |
| Junior radiologists | 0.802 (0.727 - 0.877) | |
| Senior radiologists | 0.802 (0.732 - 0.873) | |


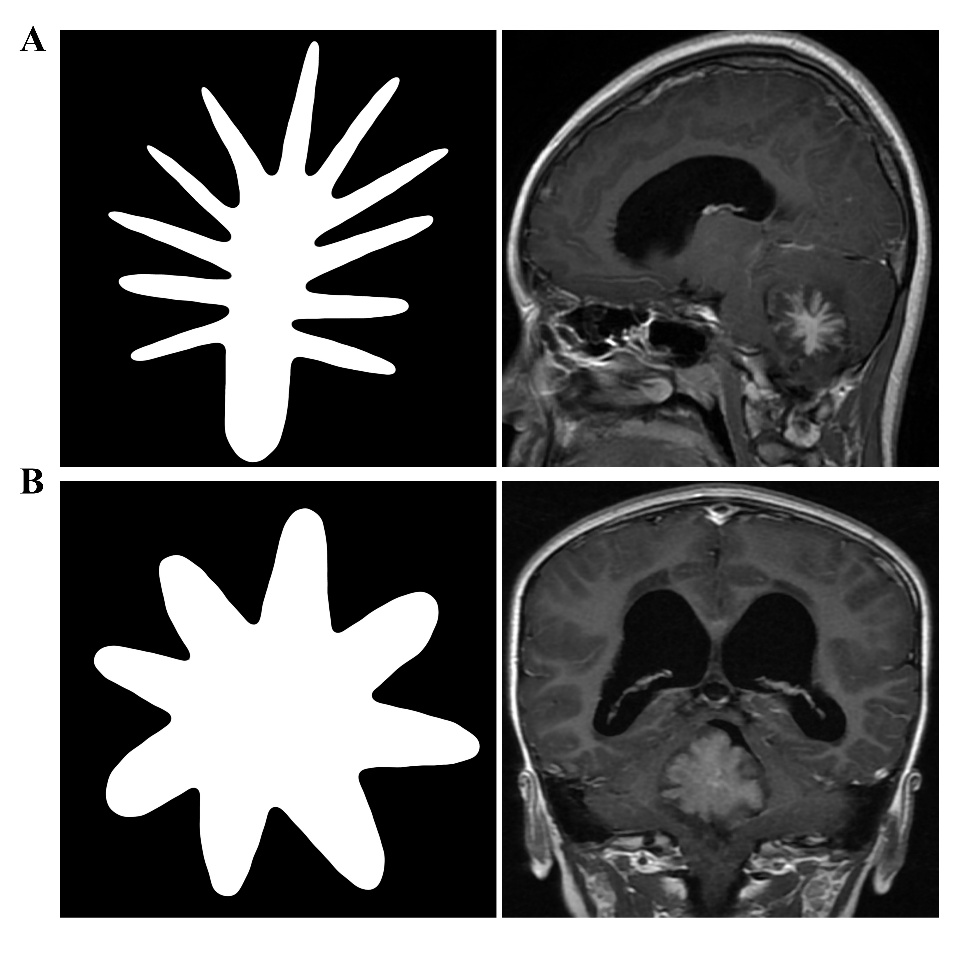


**Figure S1. Schematic diagrams of the sea anemone sign.** (A) Schematic diagram (left) and representative case (right) of the sea anemone sign with a central enhancing trunk. (B) Schematic diagram (left) and representative case (right) of the sea anemone sign without a central enhancing trunk.


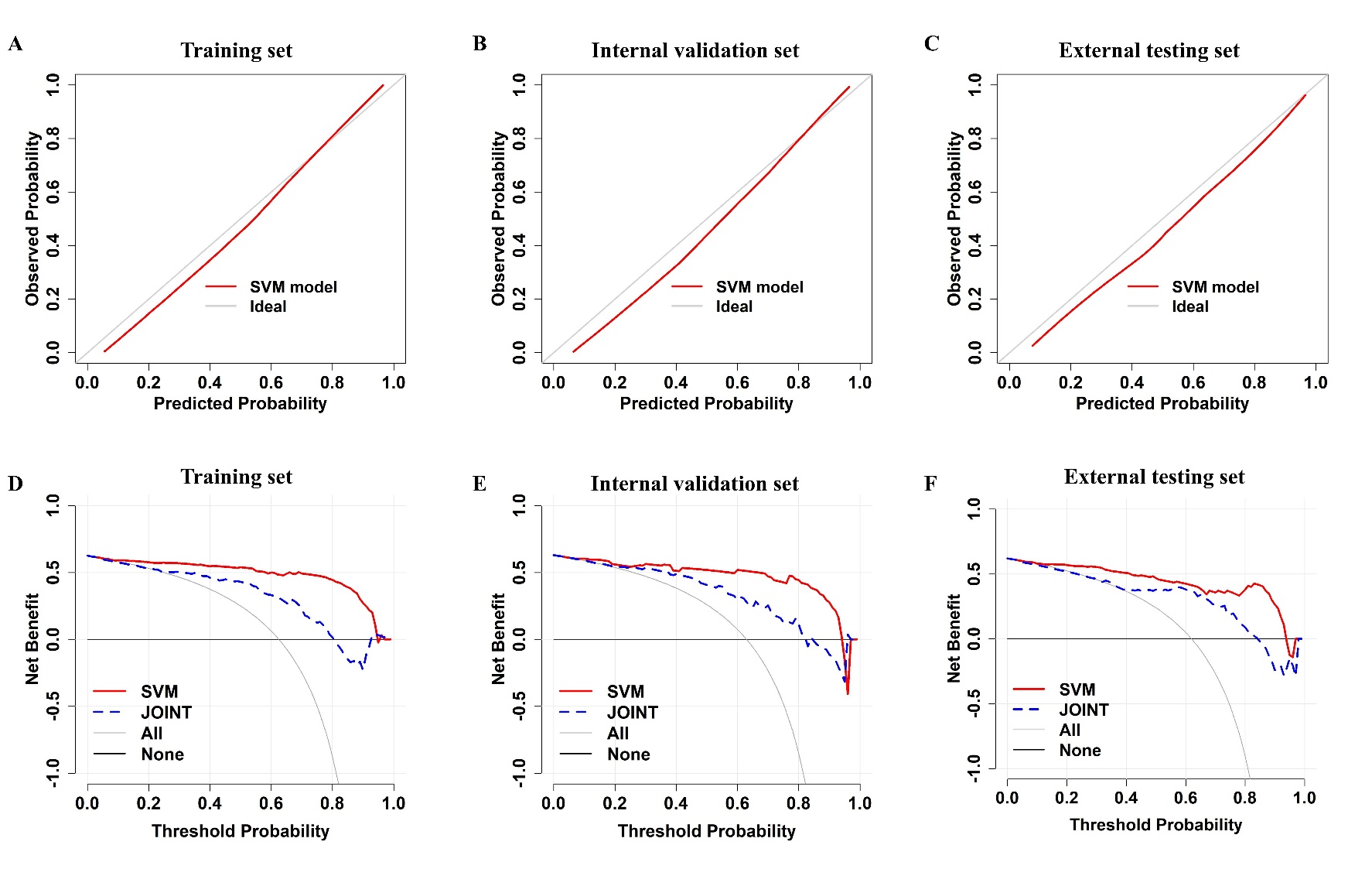


**Figure S2. Calibration** **(A-C) and** **decision curve analysis curves (D-F) of SVM Model in the training, internal validation and external testing sets. Abbreviations:** SVM = support vector machine.


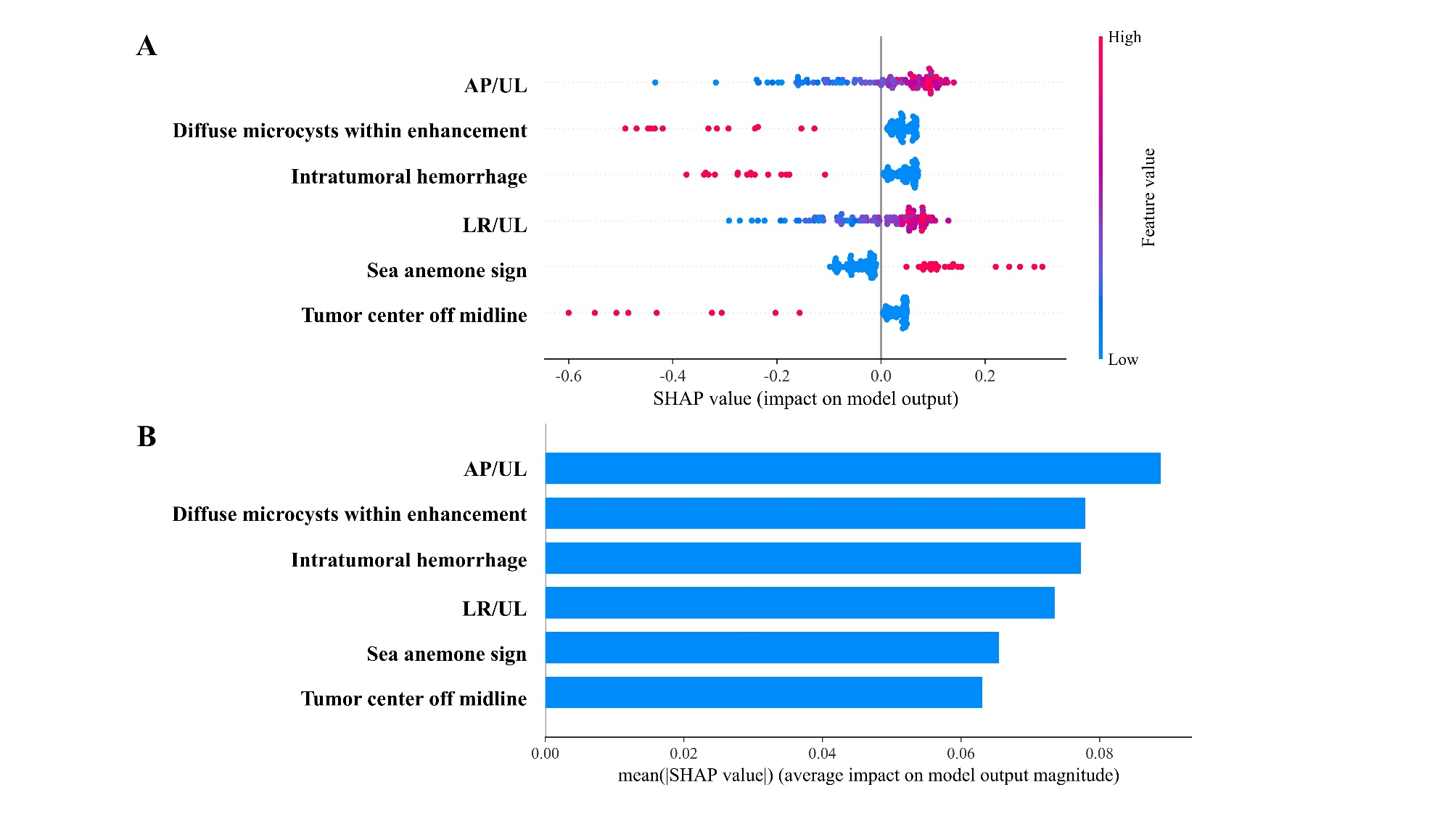


**Figure S3. SHAP value of features for SVM model prediction. Abbreviations:** LR/UL = the ratio of the left-right diameter to the upper-lower diameter, AP/UL = the ratio of the anterior-posterior diameter to the upper-lower diameter
